# Fallopian Tube Sperm Perfusion in Treatment of Nontubal Subfertility: Is It Crucial Step prior to ART?

**DOI:** 10.5402/2011/160467

**Published:** 2011-11-30

**Authors:** Maher Shams eldeen Hassan

**Affiliations:** Mansoura Faculty of Medicine, Mansoura University, Egypt

## Abstract

*Objective*. To evaluate the efficacy of double Fallopian tube sperm perfusion in comparison with single-sperm perfusion in patients with nontubal subfertility undergoing controlled ovarian stimulation. *Study Design*. Sixty-six patients undergoing standard ovarian stimulation regimen were randomized to receive either single-sperm perfusion group 1 (*n* = 33) or double-sperm perfusion group 2 (*n* = 33). The same insemination method was performed in subsequent cycles if the patient does not become pregnant in the first one. A maximum of three cycles was performed. Fallopian tube sperm perfusion was carried out with pediatric Foleys catheter, which prevents reflux of sperm suspension. Semen was prepared by a classical swim-up technique. *Results*. A total of 133 cycles performed 68 single FSP cycles and 65 FSP cycles. There were group, 19 clinical pregnancies (29.2% per cycle) of which 16 ongoing pregnancies (24.6% per cycle) were obtained. These differences were statistically significant. The prevalence of multiple pregnancies, abortions, and ectopic pregnancies was similar in both groups. *Conclusion*. The results of this study indicate that there is a significant improvement of pregnancy rates in patients with nontubal subfertility when treated with double-sperm perfusion after controlled ovarian stimulation in comparison with single-sperm perfusion. Double-sperm perfusion is simple, easy to perform, inexpensive, and convenient for the patients with nontubal subfertility before adoption of other methods of assisted reproduction.

## 1. Introduction

Artificial insemination in conjunction with ovarian stimulation is usually offered to infertile couples when the woman has patent Fallopian tubes prior to other assisted reproductive methods [1]. Ovarian stimulation may correct subtle problems of ovulation, increase the number of oocytes available for fertilization, and enhance the accuracy of timing of insemination. After artificial insemination, a higher number of motile spermatozoa with normal forms are deposited close to the site of fertilization. Moreover, sperm preparation removes leukocytes and dead spermatozoa from the semen sample; they generate free oxygen radicals and reduce the functional capacity of intact spermatozoa [2]. Intrauterine insemination is simple, noninvasive, and cost effective. The IUI technique is based on the intrauterine injection of 0.2–0.5 mL of sperms suspension without flushing the tubes. Single IUI during stimulated cycle achieves a 10–15% pregnancy rate per cycle [3]. Different ways have been explored to improve the success rate. Double IUI has been shown to improve the success rate. Double IUI has been shown to increase the pregnancy rate when compared to single IUI [4, 5]. Fallopian tube sperm perfusion (FSP) is another simple noninvasive method of delivering sperm to Fallopian tubes. It is based on pressure injection of 3 up to 5 mL of sperm suspension with the attempt of sealing the cervix to prevent sperm reflux [6, 7]. 

There is firm evidence that FSP gives rise to higher pregnancy rates than standard IUI in couples with subfertility and therefore should be advised in these couples [8, 9] as shown by Cantineau et al. [10]. However, unlike double IUI explored I, the efficacy of double FSP has not been yet explored. 

The objective of this study was to evaluate the efficacy of FSP either by single-sperm perfusion or double-sperm perfusion in patients with nontubal subfertility undergoing ovarian stimulation. 

## 2. Materials and Methods

Patients with non-tubal subfertility attending the Infertility Unit at the Department of Obstetrics and Gynecology Mansoura University Hospital were invited to participate in the Patients had to fulfill the following inclusion criteria (1) age of women <35 years, (2) duration of infertility >2 years, (3) normal hysteroscopy and laparoscopic finding with regular uterine cavity and patent tubes, and (4) normal semen parameters according to the WHO standard positive postcoital test. Every patient was extensively counseled and gave informed consent prior to participating in the study. 

All patients underwent ovarian stimulation with standard protocols were for ovarian stimulation with Merional (IBSA) starting on cycle day 3 baseline, and transvaginal scanning was performed, and when no ovarian cyst 150 IU of FSH was titrated according to ovarian response and monitored, and by transvaginal scanning when the follicle was >18 mm in diameter and when there were not more than 3 follicles >16 mm in diameter, 10000 I choriomon (IBSA) was given intramuscular in the first treatment cycle, and patients were randomized on day of HCG administration according to computer-generated randomization list, to undergo single or double FSP. The same insemination method was performed in subsequent cycles if they did not become pregnant in the first one; a maximum of three cycles was performed; single FSP was performed 36 hours after HCG administration, whereas double FSP was performed 18 and 42 hours after HCG semen was collected by masturbation after 4 days of ejaculatory abstinence and was allowed to liquefy at 37°C for 20 minutes before processing semen was prepared by classical swim-up technique using Ham and then centrifuged at 1500 rpm for 10 minutes. The supernatant was discarded, and the pellet was resuspended in 0.5 mL of the medium, then 1 mL of the medium was gently layered over the specimen. The tube was inclinated at angle 45 and incubated for 60 minutes at 37°C. It was then returned to the upright position, and the upper most 0.5 mL was removed and resuspended in 4.5 mL of Ham's F10. The total number of motile spermatozoa in the inseminate I was assessed; Fallopian tube sperm perfusion was performed using pediatric Foley's catheter. 

The luteal phase was supported by the furthest doses of 200 mg daily progesterone (prontogest). Pregnancy test was performed 2 days from the missed period. A transvaginal ultrasound was arranged to confirm the intrauterine pregnancy and to determine the number of gestational sacs after a positive pregnancy test. Only clinical ongoing pregnancies were considered in this study; clinical pregnancy is diagnosed by presence of fatal heart beat on ultrasound examination or products of conception of abortions. 

## 3. Statistical Analysis

Continuous data were expressed as mean ± SD, and statistical significance between the two groups was determined using the Student's *t*-test; categorical data were compared using the Fishers exact or chi-square test where appropriate differences were considered statistically significant at *P* < 0.05; statistics were done using SPSS software program version 9. 

## 4. Results

A total of 66 consecutive eligible patients underwent 133 stimulated cycles and proceeded FSP. Thirty-three patients were assigned to receive single FSP and 33 double FSP. Sixty-eight single FSP cycles and 65 double FSP cycles were completed. There were no differences between the two groups with regard to the age of women, duration of infertility, and body mass index (BMI) (Table 1). As regards stimulation characteristics, the FSH total dose, the duration of FSH administration, and the number of follicles >16 mm were comparable for the two groups. There was no significant difference in the mean inseminated motile sperm count between the two groups (Table 2). There were 10 clinical pregnancies in the single FSP-group of which 8 were ongoing. In the double FSP group, there were 19 clinical pregnancies of which 16 were ongoing. The differences between the two groups, both in pregnancy per cycles and patients, were statistically significant. No significant differences were found in the prevalence of multiple pregnancies or abortions between the two groups. All multiple pregnancies were twins. In both groups no case of ectopic pregnancy was observed (Table 3).

## 5. Discussion

Repeated insemination during the previously periovulatory period may improve the chance of pregnancy by increasing the number of spermatozoa inseminated enhancing the fertilizing capacity of spermatozoa to fertilize oocytes that are released over a period of at least several hours [11]. Silverberg et al. [4] first reported a dramatic increase in the pregnancy rate per cycles after double IUI when compared to single IUI (54.2% versus 8.7%) in patients having ovulatory dysfunction, unexplained infertility, or mixed factors. However, Ng et al. [12] and Kahn et al. [13] found similar pregnancy rates after single and double IUI, whereas better results for double IUI were found by Ragni et al. [5]. Therefore, it is still controversial whether double IUI achieves better pregnancy rate than single IUI; on the other hand, unlike double IUI, the efficacy of double FSP compared with single FSP has not been investigated yet. Thus, the present study aimed to investigate that issue with patients with nontubal subfertility. The volume of inseminate used in single and double ranges was from 0.2 to 0.5 mL [3]. A large inseminate volume (3–5 mL) has been employed during FSP. But part of the inseminate is flushed through the Fallopian tubes towards the pouch of Douglas, maintaining a larger number of spermatozoa in the tubes [15]. Reflux of the inseminate is prevented by an Allis Clamp on the cervix [6], cervical double nut bivalve speculum [14], intrauterine injector with inflatable balloon or pediatric Foley's catheter [15, 16]. In the present study, we simply used the pediatric Foley's catheter balloon system in single and double FSP which were easily performed in all patients and with no case of inseminate reflux observed. In most of the previous studies, FSP was compared with standard IUI, and different results were obtained; in some studies, the pregnancy rate was significantly higher than IUI [6, 7, 10, 14]. However, despite these controversial results, a recent Cochrane meta-analysis and systemic review has that in patients with non-tubal fertility, FSP proved to give rise to higher pregnancy rates than IUI and therefore should be advised in these patients [8, 9]. 

In the previous studies, the pregnancy rate per cycle achieved in couples with unexplained infertility after ovarian stimulation when FSP was used has been reported to be in the range of 8.6–40% [6, 7, 10, 14]. The results reported in this study concerning single FSP group were comparable. In this group, the pregnancy rate per cycle was 14.7% and was 30.3% per patient. The variation in the results among the different studies may be attributed to the different stimulation protocols or the types of catheters used for insemination.

Regarding double FSP, our results indicated that both clinical and ongoing pregnancy rates per cycle (29.2 and 24.6, resp.) were significantly higher than those with single FSP (14.7% and 11.7%, resp.). Pregnancy rate per patient was also significantly higher in the double FSP group. Based on medline search of studies published in the last 15 years as well as thorough review of the references cited in these identified published series and of the literature, our study is the first to evaluate the efficacy of doubles in the treatment of nontubalfertility, and accordingly no similar studies were found for comparison; however, the same principle of totally injecting large inseminate volume, used in our study by double FSP, has been recently evaluated by another method called intrauterine tuboperitoneal insemination, in which 10 mL of the inseminate was used. This method was found to give significantly higher pregnancy rates than single FSP (29.4 versus 17.6). 

The better results of double FSP may be explained by that repeated insemination achieves the presence of at the site of higher concentration of motile spermarzoa around the oocytes. 

At the site of fertilization as well as in the pouch of Dougals furthermore, repeated insemination under pressure may be necessary for achievement of perfusion spill in normal tubes or tubes with minimal adhesions. This pressure is also necessary, in midfollicular phase, and before ovulation, the endometrial glandular lumen of the Fallopian tube is narrowed by some quantities of gland by partial tubal luminal obstruction due to the presence of tubal ostium membranes. This has shown to be useful procedure in patients with subfertility and may be attempted in such patients before moving on towards much more expensive methods of assisted reproductive techniques.

## 6. Conclusion

The results of this study indicated that there is a significant improvement of pregnancy rates in patients with non-tubal subfertility when treated with double FSP after controlled ovarian stimulation in comparison with single FSP. Double FSP method is simple, easy to perform, inexpensive, and convenient for the patients. Trails of this method are useful before the adoption of other expensive assisted reproductive techniques. However, its efficacy regarding other factors as endometriosis or abnormal semen parameter remains to be investigated.

## Figures and Tables

**Table 1:** Comparison of demographic data.

	Single FSP (*n* = 33)	Double FSP (*n* = 33)	*P *value
Age (years)	30.8 + 4.6	31.5 + 4.1	NS
Duration of infertility (years)	3.9 + 1.8	3.7 + 2.2	NS
BMI (Kg/m^2^)	26.6 = 2.7	25.1 + 2.4	NS

Values are given as mean + SD.

FSP: Fallopian tube sperm perfusion.

BMI: Body mass index.

NS: Not significant.

**Table 2:** Comparison of stimulation and insemination characteristics.

	Single FSP (*n* = 33)	Double FSP (*n* = 33)	
FSH (IU)	1296 = 415	1207 + 502	NS
FSH duration (days)	11.8 + 2.7	10.5 + 2.4	NS
No. of follicles >16 mm	1.7 + 0.8	1.8 + 0.8	NS

Inseminated motile sperm			
Count × 10			
1st inseminate	12.6 + 4.1	13.8 + 3.3	NS
2nd inseminate	—	9.7 + 1.9	

**Table 3:** Comparison of treatment outcome.

	Single FSP (*n* = 68 cycles)	Double FSP (*n* = 65 cycles)	*P *value
Clinical pregnancy rate/cycle (%)	14.7 (10/68)	29.2 (19/65)	<0.01
Clinical pregnancy rate/patient (%)	30.3 (10/33)	57.6 (19/33)	<0.05
Ongoing pregnancy rate/cycles (%)	11.7 (8/68)	24.6 (16/65)	<0.01
Ongoing pregnancy rate/patient (%)	24.2 (8/33)	48.5 (16/33)	<0.05
Multiple pregnancy rate (%)	10 (1/10)	10.5 (2/19)	NS
Abortion rate (%)	20 (2/10)	15.8 (3/19)	NS
Ectopic pregnancy rate (%)	0	0	

FSP: Fallopian tube sperm perfusion.

NS: not significant.
